# Supplementary material for: Generalist Predators Shape Biotic Resistance along a Tropical Island Chain
Source: Plants (Basel). 2023 Sep 18;12(18):3304. doi: 10.3390/plants12183304 (PMC10536499; doi:10.3390/plants12183304)
Supplement: Supplementary file 1 [file plants-12-03304-s001.zip › plants-2600832-supplementary.pdf]

Supplementary Table S1. Key features of the FAW predation trials (top) and cassava mealybug natural enemy surveys (bottom) in different island settings Indonesia. For mealybug surveys, cassava fields were visited in 5 different Indonesian islands over 2017-2018, with the island of Timor also comprising the sovereign state of East Timor or Timor Leste. For FAW predation trials, four types of assays were carried out during the course of 2022 i.e., egg predation (on plant canopy), larval predation (on plant canopy and soil surface) and pupal predation (on soil surface). A first round of assays was not performed in the two islands within the Banda archipelago i.e., Besar and Ai. Approximate size and maxim. altitude (in meters above sea level, masl) of each island are indicated, as well as the exact timing of the different assays or surveys.

Fall armyworm

**Experimental assay**	**Research site**			
	Seram (17,100 km^2, 3,027 masl)	Ambon (743 km^2, 903 masl)	Banda-Besar (26 km^2, 640 masl)	Banda-Ai (3 km^2, 98 masl)
Egg predation (plant canopy)	1. –<br>2. Oct. 11<br>3. Nov. 9	1. –<br>2. Sept. 27<br>3. Oct. 20	1. –<br>2. Oct. 4<br>3. Nov. 21	1. –<br>2. Oct. 2<br>3. Nov. 19
Larval predation (plant canopy)	1. Aug. 11<br>2. Sept. 7<br>3. Oct. 11	1. June 14<br>2. Sept. 16<br>3. Oct. 18	1. –<br>2. Oct. 4<br>3. Nov. 21	1. –<br>2. Oct. 2<br>3. Nov. 19
Larval predation (soil surface)	1. Aug. 11<br>2. Sept. 7<br>3. Oct. 11	1. Aug. 27<br>2. Sept. 27<br>3. Oct. 25	1. –<br>2. Oct. 4<br>3. Nov. 21	1. –<br>2. Oct. 2<br>3. Nov. 19
Pupal predation (soil surface)	1. Sept. 7<br>2. Oct. 10<br>3. Nov. 10	1. Sept. 3<br>2. Oct. 3<br>3. Nov. 10	1. –<br>2. Oct. 4<br>3. Nov. 21	1. –<br>2. Oct. 2<br>3. Nov. 19

Cassava mealybug

	**Islands**				
	Sumatra	Java	Timor	Flores	Lombok
Size	473,481 km^2	128,297 km^2	45,783 km^2	15,531 km^2	4,739 km^2
Maximum altitude	3,805 m	3,676 m	2,963 m	2,370 m	3,726 m
Time-span	Oct. 2018	Oct.-Nov. 2017, Sept.-Oct. 2018	Oct. 2017	Oct. 2017	Oct. 2017
Districts	Lampung Tengah, Lampung Utarah, Pesawaran	Lebak, Bogor, Pati, Malang, Mojokerto	Kupang, Timor Tengah Selatan	Nagekeo, Ende, Maumere, Talibura	North Lombok
